# Market segmentation and consumer motivations in protected natural parks: A study from Spain

**DOI:** 10.1371/journal.pone.0296199

**Published:** 2024-01-02

**Authors:** Mauricio Carvache-Franco, Conrado Carrascosa-López, Wilmer Carvache-Franco

**Affiliations:** 1 Universidad Espíritu Santo, Samborondón, Ecuador; 2 Management Department, Universitat Politècnica de València, Valencia, Spain; 3 Facultad de Ciencias Sociales y Humanísticas, Escuela Superior Politécnica del Litoral, ESPOL, Guayaquil, Ecuador; Fiji National University, FIJI

## Abstract

There has been an increase in the interest of tourists in the environment and enjoying it in recent years. This research aims to: a) Identify the motivational dimensions in the protected natural parks and b) Analyze the different segments in the protected natural parks. The research was conducted in the Posets-Maladeta protected area in Spain. The sample comprises 422 surveys got in situ. Data analysis involved the execution of factor analysis and non-hierarchical K-means segmentation. The findings suggest the presence of nine motivational dimensions in the protected natural park studied: self-development, interpersonal relationships, security measures, establishing personal bonds, escape, ego-defensive function, nature, entertainment, and rewards. In addition, within this protected natural park is confirmed the presence of two distinct segments: one focused on nature and the other encompassing multiple motives. The results contribute management guidelines for natural park managers for the benefit of communities and visitors. Additionally, this work can serve as a valuable addition to the academic literature concerning ecotourism.

## 1. Introduction

Nature ecotourism has grown rapidly in recent years, it has experienced a growth rate of 5% surpassing general tourism by threefold [1]. The number of tourists that protected areas receive has reached a peak in the last decades [2]. This is due to the fact that tourists are increasingly environmentally conscious, which serves as a greater motivation to visit various tourist attractions and engage in various nature-related activities [3]. Likewise, tourists want experiences that are meaningful in their lives, for instance, engaging in activities like collaborating with local communities, learning about ecosystems and be a participant in the conservation of natural resources [4]. In this sense, ecotourism and nature areas are important destinations for environmental protection, environmental education, recreation, and local employment generation [5]. Similarly, it is important that natural landscapes are protected within national parks [6]. It is also important to take into account the reduction in forest cover, which is the area of land covered by forests [7].

It is essential when studying the behavior of tourists to take into account the motivations. This determines the different characteristics of the trips, the tourists’ reasons for traveling and the general satisfaction that tourists have had on the trip [8]. In this sense, ecotourists have profiles, motivations, and travel behavior characteristics that are different from those of other tourists [9, 10]. Therefore, among the various methods, motivational segmentation has been regarded as the most precise method for identifying distinct groups of tourists in protected areas [11]. In addition, several empirical investigations have concluded that motivations are basic elements in segmentation in tourism, for example, [12].

Demand segmentation has been employed to discover diverse market niches for the promotion of various tourism products and services [13]. For this reason, this tool is used as an important strategy in tourism research [14]. The segmentation technique has certain significant advantages for tour operators worldwide strive to guarantee that consumers receive exceptional experiences according to their expectations [15]. In this sense, demand segmentation serves as a technique that has become essential, helping managers of tourism companies to find visitor motivations, identify different information channels and focus on groups of potential customers. In this way, sustainable development is improved [16]. Unfortunately, there are few studies on the segmentation of demand in Protected Natural Parks [17].

In the mountainous area in the northeastern sector of the Aragonese Pyrenees in Spain is the Posets-Maladeta Natural Park. Here are some of the tallest summits of the Iberian Peninsula. It is a very touristic area with a varied natural diversity appreciated by its visitors. In this context, it is important to understand the distinctive attributes of each segment of ecotourism and nature. This article aims to: a) Identify the motivational dimensions in the protected natural parks and b) Analyze the different segments in the protected natural parks. This study will allow the elaboration of more efficient strategies for each segment, bringing advantages to both the destination and the community.

## 2. Literature review

### 2.1. Protected natural parks motivations to visit

Motivation can often cause a psychological imbalance in visitors, but this can be improved with a good travel experience [18]. Motivation refers to an internal psychological need that tourists have that allows them to achieve certain desires [19]. If we study the motivations, we will understand the way in which tourists choose, their preferences and the needs that travelers have [20]. Motivations make up the requirements a person possesses to engage in a tourist activity [21]. In addition, the motivations direct and integrate the behavior of the traveler and the tourist activity [22]. Therefore, motivations play an essential role in decision making [23]. Tourists have different motivations for visiting various nature attractions and destinations [24]. In this sense, tourists know what their motivations are and relate them to the different experiences they have had in the destination [25]. Regarding ecotourism, an ecotourist is identified as a "green tourist" who likes nature tourism, seeks to carry out activities in nature and is motivated to protect the environment [26].

Among the studies of motivations in nature tourism and natural parks, we find Pearce & Lee [26], who pointed out that the motivating factors of tourists were relaxation, escape, improvement of relationships and self-development. While Jang & Wu [27] noted that the drivers were relaxation, knowledge seeking, and family togetherness, the most frequent drivers were cost, natural and historical settings, security, facilities, and accessibility. Kim Lian Chan & Baum [28] found that the motivations for ecotourism and nature were cohesion, escape, healthy activity, and gaining knowledge about nature. Instead, Kruger & Saayman [29] found the reasons for visiting a national park: to go through nature, to seek knowledge, to take photographs, to enjoy the park’s attributes, to escape and relax, and for nostalgia.

Panin & Mbrica [30] found four groups: nature, cultural and educational activities, social activities, and physical activities and sports. Lee et al., [31] discovered seven factors that drive motivation; interpersonal relationships, self-development, reward, escape, building personal relationships, defensive ego function, and admiration of nature. In another study, Iversen et al., [32] identified five dimensions in motivation: novelty, status, recreation, active nature, and social engagement. At the same time, Xu & Chan [33] found: relaxation and knowledge, Self-improvement, seeking an escape from everyday life, information and convenience, destination scenario, and various activities for fun.

Kamri & Radam [34] found four motivational factors for visiting a national park: social travel, challenge hike, nature tour, and getaway. Carvache-Franco et al., [35] identified six motivational dimensions including: interpersonal relationships and ego defense function, self-development, escape, establishing personal bonds, reward, and appreciation of nature. Chow et al., [36] found that the main motivations: relaxation, escape from everyday life, and mental and physical health. In contrast, the study by Choi G et al., [37] found three factors: nature, healing (healthy and escape), and cohesion. In another marine fauna protected reserve in Ecuador, the authors Carvache-Franco et al., [38] identified the motivations for visiting the destination: marine nature, escape and personal defense, and building personal relationships.

To know the motivations for protected natural parks, the following research question is posed:

RQ1: What are the motivational dimensions in the protected natural parks?

### 2.2. Segmentation in the protected natural parks

Segmentation holds that the market is divided into subgroups of visitors with different needs and preferences [39]. In this sense, segmentation can find tourist groups with specific characteristics, provide more targeted tourist packages, enhance destination benefits and formulate more effective policies [40]. For this reason, this tool has emerged as the primary approach for determining the target audience, efficiently including the use of resources and proposing various competitive strategies [41].

By segmenting the demand, a tourist destination can obtain numerous benefits, including a competitive advantage [42]. Therefore, the identification of market segments goes beyond communication and advertising and indicates a transition from marketing to organizational management [43]. In addition, this tool helps to determine the kind of tourist services, products and experiences [44]. Thus, segmentation allows destination managers and businessmen to better position destinations and establishments, managing to attract many tourists [45].

There are several ways to segment tourists: one of them is segmentation by demographic characteristics [46], another is by types of activities [47], by tourist spending [48], by the benefits found [49] and by motivations [50]. Motivation is one of the most commonly employed methods for segmentation [51–53]. Therefore, segmenting tourists according to motivations allows companies that offer tourist services to create products and services with a high percentage of preference and well valued in destination markets [54].

Regarding ecotourist studies on segmentation in protected areas, Perera et al., [54] found four different types of tourists: hikers, ecotourists, selfish and adventure tourists. In contrast, Cordente-Rodríguez et al., [55] found two groups: Nature, those with a single motivation to relish nature´s beauty, and Multiple motives, who combine several motivations at the same time. Sheena et al., [56] identified three groups: "Hard", who sought to carry out highly demanding activities with a desire to acquire knowledge. The "Structured" are comparable to the "Soft Ecotourist" group in that they prefer the services they receive and their strong desire for the learning fact. "Soft ecotourists" with little motivation for physical activities and like guided nature walks. Barić et al., [57] detected three groups, naturalists who want to enjoy nature. Escapists, individuals seeking an escape to overcome feelings of loneliness and ecotourists, who enjoy nature, novelty, interest in learning and experiences.

Neuts et al. [58] located four segments: Bear Watchers, who are driven by the desire to observe bears, together with the excursion to the waterfalls. Landscape lovers, whose interest was only landscapes. Tourist groups organized and motivated by the landscape and whale observing. Finally, the active explorers, driven by features of the landscape, as well as the observation of birds and bears. Another study by Jeong et al. [59] identified four segments: "Tourists who like both nature and cohesion", "Nature enthusiasts tourists who enjoy nature", "Passive tourists who only like nature" and "Tourists who want it all". Phan & Schott [60] found four segments: "Enthusiasts" who exhibit high motivation for learning and experiencing. The "passive visitors" who have low levels of motivation for learning and experiencing. The "active learners" who display a high level of motivation for learning and a very low level for experiencing. “Novelty seekers”, who have a high level of motivation due to experiencing and a low level due to learning.

Another of the studies was that of Taczanowska et al., [61] discovered four segments: the first group was driven by the desire for recreation and admiration for the mountain scenery. The second group were contemplative tourists and not consumers. The third group was further categorized into two subgroups: occasional and fitness visitors. The motivation of the fourth group stemmed from nature and the landscape. According to Choi et al., [37] their study identified four segments: general tourists, nature-seeking tourists, nature-seeking cohesion tourists, and wellness-seeking tourists. In contrast, Constantin et al. [62], selected four segments: nature, cultural tourists, leisure and eclectic tourists.

Regarding the target market, naturists are those who solely seek motivations related to nature, such as appreciating and learning from it. Several authors supported this, including Cordente-Rodríguez et al. [55], Barić et al. [57], Taczanowska et al. [61], Choi et al. [37], and Constantin et al. [62]. On the other hand, the target market for multiple motives consists of individuals with high motivations across all activities to be carried out at the destination. In other words, it is a segment with multiple motivations simultaneously. Several authors affirmed this, including Cordente-Rodríguez et al. [55], Taczanowska et al. [61], and Phan T.T.L & Schott C [60].

In order to know the segments in the protected natural parks, the subsequent research question is as follows: RQ2. What are the segments in the protected natural parks?

## 3. Methodology

### 3.1. Study area

The Posets-Maladeta Park is situated in the heart of the Pyrenees region, flanked by the Posets and the Maladeta masses. It is crowned by the Posets Peak (3375 m) and the Aneto summit (3404 m). Its landscape originated in the Quaternary glaciation in the Ice Age, where many U-shaped valleys were formed. Thirty thousand years ago, today’s valley was covered in ice and has not stopped going backward. Although the last remains of glaciers are protected under the figure "Natural Monuments of the Glaciers of the Pyrenees," they are at risk of disappearing in a few years. A consequence of the glaciers’ relapse is the appearance of the glacial origin mountain lakes, called "ibones" by the locals. In addition, the area contains several intersecting valleys created by the Esera, the Noguera Ribagorzana, and the Cinqueta rivers.

The park’s richness is based on the height difference, approximately 2000 meters, from the lowest part to the Aneto summit. We can find impressive birds such as griffons, bearded vultures, golden eagles, and endemic-protected species such as the grouse. Its flora uniqueness contains several endemic flowers, the most known of which is the edelweiss flower.

According to Spanish legislation, the Posets-Maladeta Natural Park possesses distinctive attributes that warrant utmost preservation efforts under the "National Park" designation. However, the area’s inhabitants refused to become a national park for three main reasons. First, it would mean prohibiting certain traditional activities, such as livestock. Second, it would mean a significant increase in their visits until they become too overcrowded and reach saturation, as in other Spanish national parks. Finally, it would mean losing autonomy in its management because the Spanish central government manages the national parks.

### 3.2. Method

The ethical approval of the study was in charge of the ESPOL Polytechnic University of Ecuador. Written informed consent was requested as part of the questionnaire. A three-section questionnaire was designed. In the first section, the sociodemographic questions of the respondents were located, included closed-ended questions with a single-choice option. The second part of the questionnaire presented the motivation scale, which consisted of 38-item questionnaire with a five-point Likert scale format, where 1 strongly disagreed, 5 strongly agreed, and the third section included satisfaction and loyalty questions. A 5-point Likert scale was used, where 1 was strongly disagree and 5 strongly agree. Previous studies on motivations, satisfaction and return in natural parks were used to prepare the reliable questionnaire [31, 32, 63]. In addition, it was analyzed by three experts in the area. A pilot test was designed for 25 tourists after which the appropriate modifications were made to make it easy to answer. Cronbach’s Alpha was used to analyze the reliability of the motivation scale, with a value of 0.93, which represents a highly reliable value.

The sample was made up of national and foreign tourists who visited the Posets-Maladeta Natural Park in Spain. The surveys were carried out from August to September 2021 to people over 18 years of age who were inside the protected natural park. The researchers used convenience sampling to answer the questions according to the availability of the tourists. The surveys were carried out by pollsters from the Polytechnic University of Valencia (Spain), who clarified any possible doubts on the part of the surveyed visitors

The sample size consisted of 422 valid questionnaires. The variability of the population was assigned 50% (p = q = 0.5). The study had a margin of error of +/- 5% and a 95% confidence level. Data collected and statistically analyzed with SPSS 22.0. First, a factor analysis was performed to reduce the items in a smaller number of factors. Factor analysis has been used in segmentation research [64–66]. A Varimax rotation was used to order the data. To find the appropriate number of factors, the Kaiser criterion was used, only factors with eigenvalues greater than 1 were considered. The suitability of the factor analysis model was assessed using the KMO index (Kaiser-Meyer-Olkin) and Bartlett’s sphericity test. Then, in the second stage, the K-means clustering method was employed, a widely-used technique in tourism segmentation research [15]. Finally, the Chi-Square analysis was utilized to identify significant differences between the groups based on satisfaction and return behavior.

## 4. Results

From the most important data of the sample, we have that 51.7% were men and 48.3 were women. 46.9% were single, while 42.4% were married. 29.9% of the sample were between 40 and 49 years old. In contrast, 20.1% of those surveyed were between 30 and 39. 45% had a university education, and 21.6% had a postgraduate education. 53.9% visited the destination in groups of 3 and 6 people, while 26.1 in groups of less than three people. 28.7% spent between 20 and 28 euros per person per day, while 27.7% spent less than 20 per day.

### 4.1. Consumer motivation in protected natural parks

Factor analysis was used to reduce the number of motivational items in a smaller number of factors to facilitate the interpretation of the results. The Varimax rotation method was used to order the information of the factors. The Kaiser criterion was used to select the factors with eigenvalues greater than 1. Nine factors were part of the solution and represented 68.16% of the total variance. All factor loadings were greater than 0.5. The Cronbach’s alpha index in the factors varied between 0.714 and 0.917. The KMO index (Kaiser-Meyer-Olkin) obtained a value of 0.896, indicating excellent suitability for the model. Furthermore, Barlett’s sphericity test was significant at <0.05, confirming the appropriateness of applying factor analysis. The results are presented in Table 1.

**Table 1:** Consumer motivations in the protected natural parks (factor analysis).

Factors	Factor Loading	Eigenvalues	Variance Explained (%)	Cronbach’s α
F1: Self-development		11.062	29.111	0.917
To understand more about myself	0.840			
To have a chance to get to know myself better	0.816			
To gain a new perspective on life	0.749			
To gain a sense of self-confidence	0.737			
To know what I am capable of	0.693			
To be independent	0.682			
To feel inner harmony/peace	0.623			
To learn about destination	0.600			
F2: Interpersonal relationships		3.557	9.360	0.801
To strengthen relationship with my family	0.748			
To reflect on past memories	0.695			
To reminisce about parents’ times	0.687			
To feel that I belong	0.687			
To contact with family/friends who live elsewhere	0.672			
F3: Security measures		2.433	6.403	0.790
To be served with security measures personnel, mask, and gloves	0.773			
To be in a destination with security, guarantee, and health	0.728			
To visit the destination with distancing measures between people	0.719			
To be in continuously disinfected accommodations and restaurants	0.693			
To be in a destination with enough outdoor space	0.548			
F4: Building personal relationships		2.053	5.403	0.837
To meet new people	0.834			
To meet people with similar interests	0.798			
To meet local people	0.635			
To experience different cultures	0.573			
F5: Escape		1.618	4.259	0.815
To be away from daily stress	0.802			
To escape the routine	0.745			
To be away from the crowds of people	0.692			
To avoid interpersonal stress	0.520			
F6: Ego-defensive function		1.557	4.096	0.839
To join social discussion	0.804			
To follow current events	0.783			
To join people’s interests	0.720			
F7: Nature		1.322	3.478	0.756
To gain a better appreciation of nature	0.840			
To be close to nature	0.802			
To learn about nature	0.726			
F8: Entertainment		1.203	3.167	0.714
To have fun	0.802			
To have fond memories	0.728			
To gain a sense of self-achievement	0.619			
F9: Rewards		1.094	2.880	0.778
To explore the unknown	0.830			
To experience new things	0.790			
To develop my personal interests	0.573			
Total variance extracted (%)			68.158	
Cronbach’s α of all items				0.930

According to the results, the first dimension was related to understanding myself and getting to know me more, knowing what I am capable of, and learning more about the destination. For that reason, this factor is called Self-development. The second factor was related to relationships with family and friends, which is why this factor has been called Interpersonal relationships. While the third dimension was related to security measures for personnel, security at destinations, and services, for which this factor has been called Security measures. On the other hand, the fourth factor was motivations related to meeting people and local inhabitants, which is why this dimension was called building personal relationships. The fifth factor was integrated by the motivations associated with escaping from the daily routine, and avoiding stress and crowds, the reason why this dimension was called Escape. While the sixth dimension was related to motivations such as joining the social discussion and the people’s interest, for which this factor was called Ego-defensive function.

On the other hand, the seventh factor was related to observing, appreciating, and learning from nature, so this dimension was named Nature. The eighth dimension comprised motivations about fun and good memories. Thus, it was named Entertainment. Finally, the ninth factor was related to experiencing new and unknown things, which is why this dimension was called Rewards. Therefore, the research question is answered: RQ1: What are the motivational dimensions in the protected natural parks?

### 4.2. Market segmentation in the protected natural parks

Non-hierarchical K-means clustering analysis was used to segment demand, maximize variation between types, and minimize variation internally, within segments. Table 2 presents the results.

**Table 2:** Market segmentation in the protected natural parks.

Variables	Group1: Nature	Group2: Multiple motives	Factor to which each item corresponds
To be close to nature	4.5	4.8	Nature
To gain a better appreciation of nature	4.4	4.7	
To learn about nature	3.6	4.3	
To visit the destination with distancing measures between people	2.6	3.3	Security measures
To be in a destination with security, guarantee, and health	2.7	3.6	
To be served with security measures personnel, mask, and gloves	1.8	2.7	
To be in continuously disinfected accommodations and restaurants	1.9	2.6	
To be in a destination with enough outdoor space	3.7	4.5	
To experience new things	3.7	4.5	Rewards
To explore the unknown	3.9	4.6	
To develop my personal interests	3.5	4.4	
To have fond memories	4.2	4.7	Entertainment
To have fun	4.3	4.8	
To gain a sense of self-achievement	3.5	4.4	
To experience different cultures	2.5	3.5	Building personal relationships
To meet new people	2.2	3.1	
To meet people with similar interests	2.1	3.3	
To meet local people	2.5	3.4	
To have a chance to get to know myself better	2.5	4.0	Self-development
To understand more about myself	2.4	4.0	
To gain a new perspective on life	2.6	4.1	
To know what I am capable of	2.9	4.4	
To gain a sense of self-confidence	2.7	4.3	
To feel inner harmony/peace	3.4	4.6	
To be independent	2.7	4.0	
To reminisce about parents’ times	1.9	3.0	
To contact with family/friends who live elsewhere	1.9	2.7	Interpersonal relationships
To feel that I belong	1.7	2.7	
To strengthen my family relationships	2.6	3.5	
To reflect on past memories	1.8	3.2	
To learn about destination	1.8	3.6	
To avoid interpersonal stress	2.6	4.3	Escape
To be away from the crowds of people	3.2	4.4	
To be away from daily stress	3.8	4.6	
To escape the routine	3.9	4.6	
To join the interest of the people	2.0	3.0	Ego-defensive function
To join social discussion	1.6	2.4	
To follow current events	1.5	2.3	

According to the results, the Nature segment was a group with high scores in the motivations related to nature and entertainment and low scores in the other motivations. So, it is a group that was only motivated by activities related to nature and entertainment. Therefore, more specific services and activities should be offered to this segment, such as those related only to nature and entertainment. Similar to studies such as Cordente et al. [55], Barić et al. [57], Taczanowska et al. [61], Choi et al. [37], and Constantin et al. [62]. At the same time, the Multiple motives segment was a group with high scores in all motivations except those related to social and interpersonal relationships. This segment is similar to the one found by several authors with high motivations in all items, such as Cordente et al. [55], Taczanowska et al. [61], and Phan T.T.L & Schott C. [60]. However, it differs from previous studies due to the low motivations for social and interpersonal interactions. Therefore, this group should be made an offer of services that include various activities such as those related to self-development, rewards, entertainment, and escape. But it includes very few social interaction activities.

A discriminant analysis was conducted to correctly classify the tourists who belonged to each segment. Motivations were used as independent variables, and segment was used as the dependent variable. The canonical correlation was high (0.836), indicating a strong association between motivations and segments. The Wilks’ lambda was low at 0.302, indicating that the model had a higher discriminant power or better explained the independent variables to the dependent variable. Furthermore, this indicator is significant

The respondents’ classification matrix was used to determine how successfully the discriminant function could work. Almost all (97.3%) of the 187 cases in segment 1 were correctly classified, representing a very high accuracy rate. Likewise, 96.6% of the 235 cases in segment 2 were classified correctly. Therefore, the tourists who were part of the two segments were correctly classified. See Table 3.

**Table 3:** Discriminant analysis result.

Function	Canonical correlation	Wilks Lamda	Sig.	Chi square	Classification	
					Segment 1	Segment 2
1	0.836	0.302	0.000	478.187	187 tourists	235 tourists
					97.30%	96.60%

Therefore, the research question is answered: RQ2. What are the segments in the protected natural parks?

### 4.3. Segmentation with satisfaction and loyalty variables

Pearson’s Chi-square test was employed to establish connections between segments and satisfaction and loyalty variables. Moreover, a 5-point Likert scale was used, with 1 representing “strongly disagree” and 5 denoting “strongly agree”. The findings are presented in Table 4.

**Table 4:** Segmentation based on satisfaction and loyalty variables.

Variable	Group1: Nature	Group2: Múltiple motives	Chi-squared	Sig.
Overall satisfaction	4.48	4.62	14,540	0.002
I have the intention of coming back to these protected areas	4.48	4.69	13,902	0.008

According to the results, the Multiple motives segment had the highest scores in the satisfaction and return variables, making it the most essential segment. Therefore, the more improvements are made in this segment, the more tourists will be satisfied and will return more to the destination. Thus, the offer of services related to self-development, rewards, entertainment, and escape must be improved.

## 5. Discussion

This article aimed to analyze the segmentation and motivations in the protected natural parks that allows the preparation of strategists that benefits the community and destinations. The Self-development motivations identified in this study are similar to the Self-development motivations found by Lee et al. [31]. They are also similar to the Self-Enhancement motivations identified by Xu J. B. & Chan S. [33]. The Interpersonal Relationships motivations found in the results of this study are similar to the Interpersonal Relationships motivations found by Lee et al. [31]. Likewise, these are similar to the Social Activities motivations found in the study by Panin B & Mbrica A. [30] and to the Social Interaction motivations found in the results of study by Iversen et al. [32]. Security Measures motivations have not been found in the academic ecotourism literature.

The Building Personal Relationships motivations found in the results of this study are similar to those found in the study by Lee et al. [31]. Likewise, these are similar to the Social Activities motivations found in the study by Panin B & Mbrica A. [30] and to the Social Interaction motivations found in the study by Iversen et al. [32].

The Escape motivations found in this study are similar to the Escape motivations found in the study by Lee et al. [31] and Jeong et al. [59]. Likewise, these are similar to the Escapism from routine life motivations found in the study by Xu J. B. & Chan S. [33] and the Getaway outing motivations found in the study by Kamri T. & Radam A. [34]. In addition, they are similar to the Escape from daily life motivations found in the study by Chow et al. [36].

The Ego-defensive function motivations found in this study are similar to those found in the study by Lee et al. [31]. But, instead, the Nature motivations of this study are similar to the Nature motivations found in several studies such as the one by [30–32, 35, 37, 59].

While the Entertainment motivations of this study are similar to the Various activities for fun motivations found in the study by Xu J. B. & Chan S.[33], in contrast, the Rewards motivations found in this study are similar to the Rewards motivations found in the study by Lee et al. [31].

Regarding the segmentation in protected natural parks, this study found the Nature segment, with high motivations for nature and entertainment, and it is similar to the Nature segment found by Cordente et al. [55] and similar to the naturalistic segment of Barić et al. [57]. It is also similar to the nature and landscape segment identified by Taczanowska et al. [61]. Likewise, it is similar to the segment of responsible tourists who seek nature analyzed by Choi et al. [37]. Also similar to the nature traveler segment of Constantin et al. [62].

On the other hand, the Multiple Motives segment presented in the results of this research has high motivations in all variables, except for motivations for social and interpersonal relationships. This segment is similar to the Multiple Motive segment of Cordente et al. [55], who found a segment with high motivations in all motivational variables. It is also similar to the non-consumer contemplative tourist segment identified by Taczanowska et al. [61], with a wide range of motivations on the part of the visitors. In addition, it is similar to the Enthusiasts segment identified by Phan T.T.L & Schott C. [60], with a high level of motivation in all factors. Therefore, the Multiple Motives segment presents low motivations for social and interpersonal relationships, which had not been found in previous studies. Therefore, this segment presents a change in behavior, probably due to the pandemic, so tourists may not want to be exposed to social interactions. The contribution of this article to the scientific literature is to find behavioral changes in the segment multiple motives related to low motivations for social and interpersonal relationships. Therefore, the effects of the pandemic reduced the motivation for social interaction on the part of tourists from the multiple motives segment.

Among the theoretical implications is that there are two segments in the protected natural parks. The Nature segment that has high motivations in aspects related to nature and entertainment, found in other studies such as that of [37, 55, 57, 61, 62]. Other studies found the Multiple Motives segment, but with high motivations in all the variables, as found in the studies by [55, 60, 61]. Therefore, low motivation for social and interpersonal relationships is a particular characteristic of the Multiple Motives segment found in this study, this being the contribution to the literature.

Among the practical implications is that the segmentation technique benefits companies related to the tourism sector. It facilitates the development of more specific and particular strategies for each segment. For example, the Nature segment can offer activities related to nature such as observing flora and fauna, landscapes. In addition to learning from nature. The Multiple Reasons segment can offer activities that offer new experiences to tourists, that can create good memories on the trip and that are different from the daily routine that tourists live. In addition, they include learning and self-development. In this sense, the administrators can plan strategies related to the motivations and specific characteristics of each segment, which allow increasing the satisfaction of tourists. Regarding public policies, government and social institutions have the capacity to devise policies and action plans for each segment found in protected natural parks, improving the conservation of natural attractions and heritage. In addition, this tool enhances service quality in each segment by providing more tailored and personalized services.

## 6. Conclusions

Tourism in natural protected parks is a growing type of tourism and is increasingly in demand. It is distinguished by providing tourist activities within nature-rich and protected areas. Consequently, tourists’ motivations are closely tied to nature, along with other factors such as novelty, escapism, entertainment, social interaction, and personal growth. In this sense, segmentation in ecotourism serves as a tool to deliver more targeted and customized services, leading to higher satisfaction and loyalty among tourists.

Protected natural parks tourism has nine motivations: self-development, interpersonal relationships, security measures, relationship building, escape, ego defense function, nature, entertainment, and rewards. Therefore, it is confirmed that tourists have several motivations in this type of tourism, surrounded by nature and conservation. In addition, there are two segments in the tourism of protected natural parks: the Nature segment, which only has motivations related to nature and entertainment. While the segment of multiple motives has several high motivations in all aspects, except the motivations of social and interpersonal relationships. Therefore, this group of tourists do not feel motivated by social interaction, this being a change in behavior compared to previous studies, which found that this segment is highly motivated in all aspects, including social interaction. Therefore, it is recommended that protected natural parks offer services with reduced social interaction. In this way, tourists can be satisfied according to their motivations.

Finally, among the limitations of the study is the temporality with which the study was carried out. As future lines of research, it is proposed to investigate changes in the behavior of tourists in protected natural parks, especially changes in social interaction.

## Supporting information

S1 Dataset (SAV)

## References

[1] Hultman M, Kazeminia A, Ghasemi V. Intention to visit and willingness to pay premium for ecotourism: The impact of attitude, materialism, and motivation. J Bus Res. 2015; 68(9):1854–61. Available from: doi: 10.1016/j.jbusres.2015.01.013

[2] Tan E, Law R. mLearning as a softer visitor management approach for sustainable tourism. J Sustain Tour. 2016; 24(1):132–52. Available from: doi: 10.1080/02508281.2019.1630168

[3] Luo Y, Deng J. The New Environmental Paradigm and nature-based tourism motivation. J Travel Res. 2008; 46(4):392–402. Available from: https://doi.org/10.1177%2F0047287507308331

[4] Balmford A, Beresford J, Green J, Naidoo R, Walpole M, Manica A. A global perspective on trends in nature-based tourism. PLoS Biol. 2009; 7(6):e1000144. Available from: doi: 10.1371/journal.pbio.1000144 19564896 PMC2694281

[5] Tao TCH, Wall G. Tourism as a sustainable livelihood strategy. Tour Manag. 2009; 30(1):90–8. Available from: 10.1016/j.tourman.2008.03.009

[6] Myga-Piątek U, Sobala M, Szypuła B. Do national parks protect natural landscapes? J Nat Conserv. 2022; 68:126229. Available from: 10.1016/j.jnc.2022.126229

[7] Blaga L, Ilieș DC, Wendt JA, Rus I, Zhu K, Dávid LD. Monitoring Forest Cover Dynamics Using Orthophotos and Satellite Imagery. Remote Sens (Basel). 2023; 15(12):3168. Available from: 10.3390/rs15123168

[8] Castaño JM, Moreno A, García S, Crego A. Psychosocial approach to tourism motivation variables involved in the choice of Madrid as a destination. Tourism Studies. 2003; 158:5–41. Available from: https://turismo.janium.net/janium/Objetos/REVISTAS_ESTUDIOS_TURISTICOS/91949.pdf

[9] Marques C, Reis E, Menezes J. Profiling the segments of visitors to Portuguese protected areas. J Sustain Tour. 2010; 18(8):971–96. Available from: 10.1080/09669582.2010.497222

[10] Weaver DB, Lawton LJ. Overnight ecotourist market segmentation in the Gold Coast hinterland of Australia. J Travel Res. 2002; 40(3):270–80. Available from: https://doi.org/10.1177%2F004728750204000305

[11] Poria Y, Butler R, Airey D. Links between tourists, heritage, and reasons for visiting heritage sites. J Travel Res. 2004; 43(1):19–28. Available from: https://doi.org/10.1177%2F0047287504265508

[12] Kim SS, Kim JH, Ritchie BW. Segmenting overseas golf tourists by the concept of specialization. J Travel Tour Mark. 2008; 25(2):199–217. Available from: 10.1080/10548400802402958

[13] Park DB, Yoon YS. Segmentation by motivation in rural tourism: A Korean case study. Tour Manag. 2009; 30(1):99–108. Available from: 10.1016/j.tourman.2008.03.011

[14] Nickerson NP, Jorgenson J, Boley BB. Are sustainable tourists a higher spending market? Tour Manag. 2016; 54:170–7. Available from: 10.1016/j.tourman. 2015.11.009

[15] Zografos C, Allcroft D. The environmental values of potential ecotourists: A segmentation study. J Sustain Tour. 2007; 15(1):44–66. Available from: 10.2167/jost572.0

[16] Lancaster P, Doole I, Lowe R. Why organisations need to understand customer behaviour. Understanding and Managing Customers; Doole I, Lancaster P, Lowe R, Eds. 2005;109–36.

[17] Kim SS, Crompton JL, Botha C. Responding to competition: A strategy for sun/lost city, South Africa. Tour Manag. 2000; 21(1):33–41. Available from: 10.1016/S0261-5177(99)00094-1

[18] Beh A, Bruyere BL. Segmentation by visitor motivation in three Kenyan national reserves. Tour Manag. 2007; 28(6):1464–71. Available from: 10.1016/j.tourman.2007.01.010

[19] Bansal H, Eiselt HA. Exploratory research of tourist motivations and planning. Tour Manag. 2004; 25(3):387–96. Available from: 10.1016/S0261-5177(03)00135-3

[20] Meng F, Tepanon Y, Uysal M. Measuring tourist satisfaction by attribute and motivation: The case of a nature-based resort. J vacat mark. 2008; 14(1):41–56. Available from: https://doi.org/10.1177%2F1356766707084218

[21] Pearce PL. The social psychology of tourist behaviour: International series in experimental social psychology. Vol. 3. Elsevier; 2013.

[22] Yolal M, Rus RV, Cosma S, Gursoy D. A pilot study on spectators’ motivations and their socio-economic perceptions of a film festival. In: J Conv Event Tour. Taylor & Francis; 2015. p. 253–71. Available from: 10.1080/15470148.2015.1043610

[23] Chikuta O, Du Plessis L, Saayman M. Nature-based travel motivations for people with disabilities. African Journal of Hospitality, Tourism and Leisure. 2017; 6(1):1–16. Available from: http://www.ajhtl.com/uploads/7/1/6/3/7163688/article_40_vol_6__1__2017.pdf

[24] Borrie WT, Davenport M, Freimund WA, Manning RE. Assessing the relationship between desired experiences and support for management actions at Yellowstone National Park using multiple methods. J Park Recreat Admi. 2002; 20(3):51. Available from: http://js.sagamorepub.com/jpra/article/view/1542

[25] Swarbrooke J, Horner S. Consumer behaviour in tourism. Routledge; 2007.

[26] Pearce PL, Lee UI. Developing the travel career approach to tourist motivation. J Travel Res. 2005; 43(3):226–37. Available from: https://doi.org/10.1177%2F0047287504272020

[27] Jang SS, Wu CME. Seniors’ travel motivation and the influential factors: An examination of Taiwanese seniors. Tour Manag. 2006; 27(2):306–16. Available from: 10.1016/j.tourman.2004.11.006

[28] Kim Lian Chan J, Baum T. Motivation factors of ecotourists in ecolodge accommodation: The push and pull factors. Asia Pac J Tour Res. 2007; 12(4):349–64. Available from: 10.1080/10941660701761027

[29] Kruger M, Saayman M. Travel motivation of tourists to Kruger and Tsitsikamma National Parks: A comparative study. South African Journal of Wildlife Research-24-month delayed open access. 2010; 40(1):93–102. Available from: https://hdl.handle.net/10520/EJC117327

[30] Panin B, Mbrica A. Potentials of ecotourism as a rural development tool on the base of motivation factors in Serbia. Sustainable agriculture and rural development in terms of the republic of Serbia strategic goals realization within the Danube region Rural development and (un) limited resources. 2014; 597. Available from: https://mpra.ub.uni-muenchen.de/58558/1/AppData/Local/Microsoft/Windows/Temporary%20Internet%20Files/Content.Outlook/OLNQIEL3/Prihvaceni%20radovi/Domaci/www.tractorpartsasap.com/John-Deere-2250-air-conditioning-parts-s/Prihvaceni%20radovi/Domaci/www.statisticbrain.com/cheese-statistics/#page=617

[31] Lee S, Lee S, Lee G. Ecotourists’ motivation and revisit intention: A case study of restored ecological parks in South Korea. Asia Pac J Tour Res. 2014; 19(11):1327–44. Available from: 10.1080/10941665.2013.852117

[32] Iversen NM, Hem LE, Mehmetoglu M. Lifestyle segmentation of tourists seeking nature-based experiences: The role of cultural values and travel motives. J Travel Tour Mark. 2016; 33(sup1):38–66. Available from: 10.1080/10548408.2014.998359

[33] Xu JB, Chan S. A new nature-based tourism motivation model: Testing the moderating effects of the push motivation. Tour Manag Perspect. 2016; 18:107–10. Available from: 10.1016/j.tmp.2016.01.001

[34] Kamri T, Radam A. Motivation of Visiting Bako National Park. Asian Journal of Quality of Life (AjQoL). 2018; 3(9):123–31. Available from: 10.21834/ajqol.v3i9.83

[35] Carvache-Franco M, Segarra-Oña M, Carrascosa-López C. Segmentation by motivation in ecotourism: Application to protected areas in Guayas, Ecuador. Sustainability. 2019; 11(1):240. Available from: 10.3390/su11010240

[36] Chow ASY, Cheng INY, Cheung LTO. Self-determined travel motivations and ecologically responsible attitudes of nature-based visitors to the Ramsar wetland in South China. Ann Leis Res. 2019; 22(1):42–61. Available from: 10.1080/11745398.2017.1359791

[37] Choi G, Kim J, Sawitri MY, Lee SK. Ecotourism market segmentation in Bali, Indonesia: Opportunities for implementing REDD+. Land (Basel). 2020; 9(6):186. Available from: 10.3390/land9060186

[38] Carvache-Franco M, Carvache-Franco W, Carvache-Franco O, Borja-Morán J. Motivations as a predictor of satisfaction and loyalty in ecotourism. Journal of Outdoor Recreation and Tourism (JORT). 2022; 37:100478. Available from: 10.1016/j.jort.2021.100478

[39] Dolnicar S, Grün B. Challenging “factor–cluster segmentation.” J Travel Res. 2008; 47(1):63–71. Available from: 10.1177/0047287508318910

[40] Ho GTS, Ip WH, Lee CKM, Mou WL. Customer grouping for better resources allocation using GA based clustering technique. Expert Syst Appl. 2012; 39(2):1979–87. Available from: 10.1016/j.eswa.2011.08.045

[41] Dolnicar S, Freitag R, Randle M. To segment or not to segment? An investigation of segmentation strategy success under varying market conditions. Australasian Marketing Journal (AMJ). 2005; 13(1):20–35. Available from: 10.1016/S1441-3582(05)70065-3

[42] Lambin JJ, Schuiling I. Market-driven management: Strategic and operational marketing. Bloomsbury Publishing; 2012.

[43] Zhang J, Marcussen C. Tourist motivation, market segmentation and marketing strategies. In: 5th Bi-Annual Symposium of the International Society of Culture, Tourism, and Hospitality Research, Charleston, South Carolina. Citeseer; 2007. p. 1–27. Available from: https://citeseerx.ist.psu.edu/document?repid=rep1&type=pdf&doi=c83405a3f476d0c7182fc7a7fecc77d24c5d0469

[44] Birdir SS. Segmentation of tourist using demographic and travel characteristics: The case of Istanbul. International Review of Management and Marketing. 2015; 5(4):221–9. Available from: https://dergipark.org.tr/tr/download/article-file/366722

[45] Tsiotsou R. Using visit frequency to segment ski resorts customers. J vacat mark. 2006; 12(1):15–26. Available from: https://doi.org/10.1177%2F1356766706059029

[46] Sung HY, Morrison AM, O’leary JT. Segmenting the adventure travel market by activities: From the North American industry providers’ perspective. J Travel Tour Mark. 2000; 9(4):1–20. Available from: 10.1300/J073v09n04_01

[47] Mok C, Iverson TJ. Expenditure-based segmentation: Taiwanese tourists to Guam. Tour Manag. 2000; 21(3):299–305. Available from: 10.1016/S0261-5177(99)00060-6

[48] Frochot I. A benefit segmentation of tourists in rural areas: a Scottish perspective. Tour Manag. 2005; 26(3):335–46. Available from: 10.1016/j.tourman.2003.11.016

[49] Sellick MC. Discovery, connection, nostalgia: Key travel motives within the senior market. J Travel Tour Mark. 2004; 17(1):55–71. Available from: 10.1300/J073v17n01_04

[50] Albayrak T, Caber M. Examining the relationship between tourist motivation and satisfaction by two competing methods. Tour Manag. 2018; 69:201–13. Available from: 10.1016/j.tourman.2018.06.015

[51] Bieger T, Laesser C. Market segmentation by motivation: The case of Switzerland. J Travel Res. 2002; 41(1):68–76. Available from: https://doi.org/10.1177%2F004728750204100110

[52] Ryan C, Glendon I. Application of leisure motivation scale to tourism. Ann Tour Res. 1998; 25(1):169–84. Available from: 10.1016/S0160-7383 (97) 00066–2

[53] Lee CK, Lee YK, Wicks BE. Segmentation of festival motivation by nationality and satisfaction. Tour Manag. 2004; 25(1):61–70. Available from: 10.1016/S0261- 5177(03)00060-8

[54] Perera P, Vlosky RP, Wahala SB. Motivational and behavioral profiling of visitors to forest-based recreational destinations in Sri Lanka. Asia Pac J Tour Res. 2012; 17(4):451–67. Available from: 10.1080/10941665.2011.627353

[55] Cordente-Rodríguez M, Mondéjar-Jiménez JA, Villanueva-Álvaro JJ. Sustainability of nature: The power of the type of visitors. Environmental Engineering & Management Journal (EEMJ). 2014; 13(10). Available from: http://omicron.ch.tuiasi.ro/EEMJ/

[56] Sheena B, Mariapan M, Aziz A. Characteristics of Malaysian ecotourist segments in Kinabalu Park, Sabah. Tourism Geographies. 2015; 17(1):1–18. Available from: 10.1080/14616688.2013.865069

[57] Barić D, Anić P, Macías Bedoya A. Combining benefit-sought segmentation and service quality gap analysis: Case study of Paklenica National Park, Croatia. Tourism: An International Interdisciplinary Journal. 2016; 64(1):7–25. Available from: https://hrcak.srce.hr/154829

[58] Neuts B, Romao J, Nijkamp P, Shikida A. Market segmentation and their potential economic impacts in an ecotourism destination: An applied modelling study on Hokkaido, Japan. Tour Econ. 2016;22(4):793–808.

[59] Jeong Y, Zielinski S, soon Chang J, Kim S il. Comparing motivation-based and motivation-attitude-based segmentation of tourists visiting sensitive destinations. Sustainability. 2018; 10(10):3615. Available from: 10.3390/su10103615

[60] Phan TTL, Schott C. Visitor responses to environmental interpretation in protected areas in Vietnam: A motivation-based segmentation analysis. Tour Recreat Res. 2019; 44(4):492–506. Available from: 10.1080/02508281.2019.1630168

[61] Taczanowska K, González LM, García-Massó X, Zięba A, Brandenburg C, Muhar A, et al. Nature-based tourism or mass tourism in nature? segmentation of mountain protected area visitors using self-organizing maps (som). Sustainability. 2019; 11(5):1314. Available from: 10.3390/su11051314

[62] Constantin CP, Papuc-Damașcan V, Blumer A, Albu RG, Suciu T, Candrea AN, et al. Profiling visitors to Romanian ecotourism destinations. Sustainability. 2021; 13(5):2958. Available from: 10.3390/su13052958

[63] Kim KH, Park DB. Relationships among perceived value, satisfaction, and loyalty: Community-based ecotourism in Korea. J Travel Tour Mark. 2017; 34(2):171–91. Available from: 10.1080/10548408.2016.1156609

[64] Formica S, Uysal M. Market segmentation of an international cultural-historical event in Italy. J Travel Res. 1998; 36(4):16–24. Available from: https://doi.org/10.1177%2F004728759803600402

[65] Kastenholz E, Davis D, Paul G. Segmenting tourism in rural areas: the case of North and Central Portugal. J Travel Res. 1999; 37(4):353–63. Available from: https://doi.org/10.1177%2F004728759903700405

[66] Johns N, Gyimothy S. Market segmentation and the prediction of tourist behavior: The case of Bornholm, Denmark. J Travel Res. 2002; 40(3):316–27. Available from: https://doi.org/10.1177%2F0047287502040003009

